# The Use of Repetitive Transcranial Magnetic Stimulations for the Treatment of Bipolar Disorder: A Scoping Review

**DOI:** 10.3390/bs12080263

**Published:** 2022-07-30

**Authors:** Medard Kofi Adu, Ejemai Eboreime, Adegboyega Oyekunbi Sapara, Vincent Israel Opoku Agyapong

**Affiliations:** 1 Department of Psychiatry, University of Alberta, Edmonton, AB T6G 2B7, Canada; eboreime@ualberta.ca (E.E.); asapara@ualberta.ca (A.O.S.); vn602367@dal.ca (V.I.O.A.); 2 Department of Psychiatry, Dalhousie University, Halifax, NS B3H 2E2, Canada

**Keywords:** repetitive transcranial magnetic stimulation, bipolar disorder, mental health, treatment

## Abstract

Repetitive transcranial magnetic stimulation (rTMS) is a noninvasive neuromodulation technique that involves the application of magnetic pulses on hyperactive or hypoactive cortical brain areas. rTMS is considered a high therapeutic tool in many neuropsychiatric conditions. Despite its wide and continuous usage for the treatment of psychiatric disorders, information about the use of rTMS in bipolar disorders is limited and not well-established in the literature. **Objectives:** This scoping review aims to explore the literature available regarding the application of rTMS for the management of bipolar disorders, to garner evidence in support of it uses in the management of bipolar disorders, and for recommendations on future clinical and research work. **Method:** We electronically conducted a data search in five research databases (MEDLINE, CINAHL, Psych INFO, SCOPUS, and EMBASE) using all identified keywords across all the databases to identify evidence-based studies. Articles were included if they were published randomized control designs aimed at the use of rTMS in the management of bipolar disorders. Overall, nine studies were eligible for this review. The search results are up to date as of the final date of data search—20 December 2020. Only full-text published articles written in English were reviewed. Review articles on treatment with rTMS for conditions either than bipolar disorders were excluded. **Conclusion:** The application of rTMS intervention for bipolar disorders looks promising despite the diversity of its outcomes and its clinical significance. However, to be able to draw a definite conclusion on the clinical effectiveness of the technique, more randomized controlled studies with well-defined stimulation parameters need to be conducted with large sample sizes in the future.

## 1. Introduction

Bipolar disorder is a chronic episodic mood illness characterized by manic episodes that come with alternating episodes of depression [1]. It has an unpredictable course and can result in deficiencies in cognition, functional, and occupational functions [1,2,3]. Bipolar disorder is among the main causes of youth disability [4] and results in an elevated rate of mortality, especially death caused by suicide [5]. Bipolar disorder is deemed to possess the highest risk of suicide when compared with all other mental health conditions [6]. Suicidal tendencies in bipolar disorder vary and are dependent upon the phase of the condition. Primarily, the suicidal behavior in the illness occurs during the mixed phase of the condition and the depression phase [7,8]. 

Individuals diagnosed with bipolar disorders have a greater prevalence of medical and psychiatric comorbidities. The lifetime prevalence of bipolar disorders is over 1% of the global population irrespective of socioeconomic status, race, or nationality, with the annual prevalence of bipolar disorder estimated at 0.6% for the U.S. population [4,9]. There is a higher prevalence of the bipolar disorder in men than in women, with the prevalence ratio between males and females being 1.1:1 [9]. According to data, the most common and strongest risk factor for bipolar disorders revolves around having a family history of the condition, and the chances increase with the degree of kinship to the affected individuals [5].

The basic step in the treatment of bipolar disorder is the confirmation of the presence of mania or hypomania. Moreover, as the approach to therapy differs for the various clinical features such as depression, hypomania, mania, mixed affective state, and euthymia, the state of the mood of the patient should be defined [1,10]. Several factors affect the therapeutic efficacy of the pharmacological and psychotherapeutic intervention in the management of bipolar disorders and should be observed to optimize efficacy [11]. These factors may include, but are not limited to, the many medical and psychiatric comorbidities, the effect of previous or current medications, and the willingness of patients to receive and adhere to treatment protocols [5].

Psychopharmacological agents are considered the first-line treatment for bipolar disorder and their therapeutic efficacy has been tested across the different phases of the illness [12]. Despite their proven effectiveness, pharmacological agents for the management of bipolar disorder present with some limitations, which become a matter of concern. Notable among them is the rate of non-response to adequate pharmacotherapy [13], the unbearable side effects with its related nonadherence and discontinuation of the medication [14,15], and the possible increased medical burden due to the different medication prescribed by clinicians to cater for the different symptoms and comorbidities of bipolar illness [16,17]. Amid these limitations, and the quest for alternative efficacious treatment intervention [17], transcranial magnetic stimulations (TMSs) have been evaluated and found as a treatment option for bipolar disorder [18].

TMS was introduced as a focal brain stimulation in 1985 as a safer and painless way of studying the central nervous system, in particular to stimulate motor cortex and to assess the human central motor pathways [19]. TMS has become a major research tool in mental health care thanks to its ability to produce explicit effects on a number of measures of brain function [20,21].

TMS is a noninvasive treatment technique in which brain networks are modulated by the application of magnetic current in the hypoactive or hyperactive cortical areas of the brain [22]. The introduction of magnetic pulses is carried out by placing an electromagnetic coil over the scalp of the patient under treatment. The magnetic pulses from the coil then penetrate the skull into the cortical region of the brain with a resultant activation of neural changes in the brain [23]. The magnetic pulse can be delivered in a repeated manner to produce a long-term change in the neural activity [24]. There can either be an increase or decrease in cortical excitability through a high-frequency application (>5 Hz) or low-frequency application of 1 Hz stimulation [25,26]. TMS when delivered in trains of repetitive pulses (rTMS) is very flexible and, depending on the brain target and frequency applied, it could produce inhibition or induce local and remote brain activity [27]. An optimum rTMS is achieved when it is delivered in a train of repetitive pulses with similar stimulus intervals [24,28].

There have been several technological advances made in the application of rTMS, hence the current generation of rTMS studies has brought to bear notable limitations in earlier clinical trials and has sought to offer solutions to them [29]. The modern generation of studies demonstrates better outcomes through higher or accelerated dosing protocols [30,31], extended treatment durations [32], patient-centered stimulation frequencies [33], and a clear outline for bilateral stimulations [34].

The application of rTMS intervention is generally considered simple, safe, and well tolerated, and requires less effort to administer [35]. A major advantage is that it presents with no serious side effects [36]. rTMS is relatively cost-effective compared with other similar treatment interventions such as electroconvulsive therapy [37]. However, rTMS application presents with mild side effects such as pain in the scalp, which disappear with a moderate increase in the treatment intensity [38]. There may also be vasovagal syncope at the beginning of the treatment, hence the patient is encouraged not to raise their head during that period. To reduce the clicking sound during the process of rTMS application, the patient can make use of earplugs [39].

rTMS was cleared for use in Canada and the United States in 2002 and 2008, respectively [40,41]. It is advocated and recommended by the National Institute for Health and Care Excellence (NICE), 2015 and sanctioned by the Food and Drug Administration (FDA) as a treatment for treatment resistant depression in the USA [42,43].

The high number of data on superficial brain stimulation for mental disorders is in the domain of rTMS intervention in patients with MDD [44]. Based on its versatility and efficacy, rTMS treatment has now been expanded to other major mental health conditions sch as bipolar disorders [44]. The proof of the therapeutic benefits of rTMS, as reviewed by some European experts [45], reflected on the analgesic effect of high-frequency (HF) rTMS treatment of the motor cortex and the antidepressant effect of high-frequency rTMS application to the dorsolateral prefrontal cortex.

rTMS has been evaluated as effective in randomized double-blind sham-controlled trials (RCT) in treating unipolar depression, although it is not certain whether the efficacy of rTMS treatment extends to the management of bipolar disorders [46]. Data from a study conducted suggest that rTMS appeared superior to sham rTMS for the management of bipolar depression over a period of 2 weeks [47], while the second study found a less significant difference in response between the rTMS and the sham group in 23 patients [48]. Although the indication for the application of rTMS in bipolar disorders is strong, the evidence available is mixed and limited.

Generally, scoping review studies evaluate the literature for the potential size and scope of available data on topics of interest [49]. In this regard, this scoping review seeks to explore the existing literature and evaluate data on current studies and their main findings regarding the use and potential efficacy of the application of rTMS across symptomatic and remitted stages of bipolar disorder.

## 2. Methods

A search strategy was developed and applied to electronically conduct a data search in five databases (MEDLINE, CINAHL, Psych INFO, SCOPUS, and EMBASE) using all identified keywords to identify eligible studies and randomized controlled trials. Key terms included: repetitive transcranial magnetic stimulations, obsessive compulsive disorder, post-traumatic stress disorders, bipolar disorders, and treatment. This search strategy is made up of search results on the use of rTMS intervention in three mental health conditions (PTSD [50], OCD [51], and bipolar disorders). However, this scoping review report and discusses only the results in bipolar disorders. Table 1 displays a sample of our search strategy on Medline Database.

## 3. Inclusion and Exclusion Criteria

Studies were included if they involved completed RCT of rTMS treatment in bipolar disorders. Again, only full-text studies published in English were included.

The exclusion criteria included studies in rTMS intervention for the management of other mental health conditions either than bipolar disorders. Studies with rTMS treatment for bipolar disorders with comorbidities were not included. Studies that assessed rTMS intervention as a combined treatment with psychopharmacotherapy or similar treatment interventions were excluded in this scoping review. Study protocols and experiments of rTMS treatment that were not meant for the management of bipolar disorders were excluded. We excluded systematic reviews and meta-analyses.

Two independent reviewers (M.K.A. and E.E.) conducted both the title and abstract screening as well as the full text screening. The review came up with relevant studies that fit the purpose of this review. Thematic classifications were carried out by the two reviewers and conflicts were resolved based on discussion between the two reviewers.

**Figure 1:**
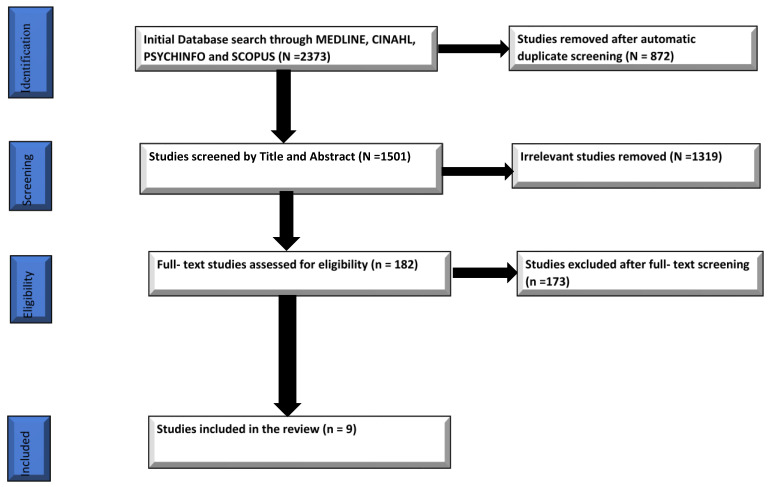
PRISMA—flow diagram summarizing search process and results.

Through the covidence platform, the search strategy realized a total of 2373 studies from all databases accessed. Covidence automatically removed a total of 872 studies as duplicates from the searched result. The remaining studies (1501) were screened against the inclusion criteria set for this review based on the title and abstract only. The screening was performed independently by the two reviewers and conflicts within the review process resolved by consensus among the two reviewers. The title and abstract screening brought the total records left for full-text screening to 182 studies. After the exclusion of a total of 1319 items. The remaining studies were full-text screened independently by the two reviewers, excluding 173 studies. Overall, 9 studies were legible for the purposes of this review. Figure 1 describes the PRISMA—flow diagram summarizing search process and results

## 4. Results

### 4.1. Summary of Results

A qualitative descriptive approach was used to categorize the reviewed studies according to name of authors, year of publication, country of publication, type of design, sample size, targeted brain regions, targeted symptoms, measurement tools, treatment duration, coil/rTMS stimulations, outcome/significant improvements, assessment and follow-up, conclusion, and side effects of the intervention. All nine studies under review applied the randomized control trial design, which includes the parallel, double-blind, and open labels methods (thus, both the health providers and study participants were aware of the intervention being given). The study sample ranged from n = 11 to n = 76. The detailed methodological data extracted and summarized are presented in Table 2.

### 4.2. Outcome Measures

Several measures were used to assess outcomes and reduction in symptom conditions. These measuring scales include HDRS, YMRS, HAMA, GAF, MADRS, and CGI-S. The safety outcome measures included adverse effect reporting, neurocognitive assessments, and vital sign assessments.

### 4.3. Frequency, Intensity of Stimulation, Duration of Treatment, and Brain Target

The data gathered from the included studies support the use of both high-frequency rTMS [52,53,54,55] and low-frequency rTMS [56,57] at 110% or 120% motor thresholds, and there was no clear superiority between the effects of the low- or high-frequency rTMS. The treatment duration of rTMS application ranged from 2 to 6 weeks for all of the included studies. Six out of the nine studies applied rTMS over the L-DLPFC [52,53,54,55,57,58], two over the R-DLPFC [56,59], with the remaining study applying rTMS to both left and right DLPFC. Despite the diversity in the choices of target to the brain regions, there seems to be no clinical difference between the target sites (left versus right DLPFC).

### 4.4. Clinical Outcomes

Of the nine studies under review, seven demonstrated significant positive outcomes and improvements in the condition of the bipolar disorder patients under study. Two of the studies failed to identify any superiority of active rTMS over sham with respect to bipolar disorder symptoms. The results suggested that rTMS treatment was safe and tolerable with no serious side-effects; however, there were a few reports of mild headache, dizziness, and scalp pain reported by some study participants, which improved during the process of application or soon after completion of the sessions of rTMS.

**Table 2:** Summary of studies using rTMS for the treatment of bipolar disorders.

Author (Year)	Country of Origin	Study Design	Number of Participants	Targeted Brain Region	Targeted Symptom	Measurement	Duration	Coil/rTMS Parameters/Stimulation Method	Outcome/Significant Improvements	Assessment and Follow-Up	Conclusion	Side Effects
Nahas et al. (2003) [57]	USA	RCT	23 participants	left prefrontal rTMS	Depressive symptoms of BPAD	HDRS, YMRS, HAM-A, GAF	2 weeks	5 Hz, 110% MT, 8 s on, 22 off, over 20 min	% change in HRSD at 2 weeks compared with day 1 of rTMS treatment	day 1 and day 10	Daily left-PFC rTMS is deemed safe in depressed BPAD patients with minimal risk of inducing mania in the patients on medications	nil
Dell’Osso et al. (2011) [59]	Chicago USA	a 1-year follow-up study (prospective study)	11 patients	right dorsolateral prefrontal cortex	Depressive recurrences Manic recurrences Mixed recurrences	HAM-D score, YMRS	3 weeks	(1 Hz), 110% MT, 300 stimuli/d for 3 weeks	The study demonstrated that achieving remission after acute rTMS treatment was predictive of maintenance of response at 1 year.	at baseline, 3, 6, and 12 months	The long-term discontinuation effects after acute rTMS treatment suggest that prompt remission is predictive of sustained benefit after 1 year	nil
M.L. Myczkowski et al. (2018) [52]	Brazil	randomized, placebo-controlled trial	50 patients	left DLPFC	symptoms of depression, anxiety, and mania, as well as rTMS adverse effects,	YMRS,	4 weeks	H1-coil 55 trains at 18 Hz and 120% MT total of 1980 pulses/day or 39,600 pulses per treatment for 8 weeks, 4 weeks of 20 daily sessions, and a follow-up of 4 weeks with no TMS sessions	There were cognitive improvements in all domains of bipolar depression. Thus, drTMS is deemed a safe antidepressant treatment for bipolar disorder patients with marked cognitive dysfunction.	at baseline, 4, and 8 weeks.	The study supports the evidence on the cognitive safety of H1-coil TMS in BD patients. Putative pro-cognitive effects of rTMS in BD were not observed	nil
L.-L. Yang et al. (2019) [53]	China	single-blind randomized controlled trial	60 patients	left DLPFC	cognitive impairment in BD participants in remission	HDRS, YMRS, MCCB	10 days	High speed figure-of-eight coil. Fifty 5 s, 10 Hz trains delivered at 110% of the MT at 30 s interval for 10 days	High-HZ rTMS treatment improves neurocognitive function in bipolar disorder	baseline clinical assessments and follow-up clinical assessments	The study demonstrated that short-term rTMS can correct cognitive dysfunction in BD patients	mild dizziness
Y. B. Yang et al. (2020) [58]	Canada	retrospective chart review	76 patients	L-DLPFC	change in clinician-rated depressive symptoms.	HRDS-21,	between 2 and 6 weeks	Magstim Super Rapid-2 device 10 Hz, 3000 pulses, 4 s trains and 26 s intertrain interval, 120% MT	BD patients are less likely to achieve clinical response compared with unipolar depression patients with high-Hz L-DLPFC rTMS application	baseline HRDS-21	The study demonstrated that BD patients are less likely to achieve clinical response compared with unipolar depression with high-frequency L-DLPFC rTMS	nil
A.L. PHILLIPS et al. (2020) [54]	USA	naturalistic retrospective patient data study	71 patients	L-DLPFC	depression response and remission rates among patients with bipolar	QIDS, HRDS	2 weeks, followed by 2 weeks of once-daily rTMS, for a total of 28 sessions with 2 daily sessions skipped	10 Hz rTMS 100% to 120% MT over 3000 pulses per session. F3 coil positioning	Patients with bipolar TRD responded equally as well as patients with unipolar TRD and showed trends for a possible early response	2 weeks, followed by 2 weeks of once-daily rTMS, for a total of 28 sessions with 2 daily sessions skipped	The study supports the use of high-frequency rTMS application over the left-DLPFC in the management of bipolar TRD	nil
Dell_Osso et al. (2009) [56]	Italy	an open-label design	11 right-handed patients	R- DLPFC	efficacy of low-frequency rTMS in bipolar disorders	HAM-D, CGI-S, YMRS	3 weeks	1 Hz and at 110% MT 8-figured coil for a total of 15 days with five trains of 60 stimuli, 300 stimuli per session	Augmentative low frequency rTMS of the right DLPFC combined with brain navigation was effective and well tolerated in a small sample of drug-resistant bipolar depressive patients,	at baseline and weekly for three weeks	Augmentative low-frequency rTMS of the right DLPFC combined with brain navigation was effective and well tolerated in a small sample of drug-resistant bipolar depressive patients	nil
F RACHID et al. (2017) [55]	Switzerland	a naturalistic clinical treatment	22 participants (10 received 5 Hz and 12 received 10 Hz)	L-DLPFC	changes in depressive symptoms and Effects of 5 Hz and 10 Hz	MADRS CGI-S	4 weeks	5 Hz or 10 Hz. rTMS over the LDLPFC. 120% to 130% of MT, 40 to 60 trains, 10 s 2000 to 3000 pulses per session	Study demonstrated clinical response, safety, feasibility, and 100% adherence rates using 5 or 10 Hz rTMS in a routine clinical setting in patients with treatment-resistant unipolar and bipolar depression	At baseline, week 1,2, 3, and 4	rTMS over the L-DLPFC was deemed safe and effective in an important subset of outpatients with a moderate to severe MDE in a naturalistic setting	nil
P.B. Fitzgerald et al. (2016) [60]	Australia	a parallel design two-arm double blind rando- mised controlled trial	49 participants	sequential manner: to the right DLPFC and then the left DLPFC in the same order in all subjects.	changes in depressive symptoms	YMRS HAMD	4 weeks	70 mm figure of 8 coils. 1 Hz R- DLPFC in a single train of 1000 pulses and L-DLPFC 10 Hz 10% RMT	No significant difference in response rates between active and sham stimulation	baseline to week 4	The study failed to detect a significant benefit of sequential bilaterally applied TMS in bipolar depression patients	nil

MT—motor threshold, DLPFC—dorsal lateral prefrontal cortex, RMT—resting motor threshold, CGI-I—clinical global impression, HRSD—Hamilton Rating Scale for Depression, YMRS—Young mania rating scale, GAF—Global Assessment of Functioning, MCCB—MATRICS consensus cognitive battery, QIDS-quick inventory of depressive symptomatology, BRMAS—Bech–Rafaelsen mania scale.

## 5. Discussions

### 5.1. Summary of Main Results

The nine studies under review in the present scoping review demonstrate that rTMS may potentially be a safe and clinically effective intervention for a difficult-to-treat condition such as bipolar disorder. The review recorded some significant improvements in the symptoms of study subjects. Overall, rTMS appeared to present with mild side-effects and was well tolerated by bipolar disorder patients.

### 5.2. Targeted Brain Regions

Out of the six studies [52,53,54,55,57,58] that assessed the effectiveness of rTMS application over the L-DLPFC, overall, their results supported the idea that L-DLPFC was a safe site and effective for the management of the symptoms of bipolar disorders. For instance, the study by Myczkowski et al. (2018) [52], with 50 participants, evaluated the clinical efficacy and safety of H1coil rTMS for bipolar disorder patients from a cognitive perspective over the left DLPFC. The H1 coil becomes fundamentally significant owing to the rate and debilitating nature of cognitive dysfunction in such patients. The study reported improvements in cognitive function in all domains of bipolar depression. The results demonstrated that that deep rTMS was a safe intervention for the management of bipolar disorder marked with cognitive dysfunction

On the other hand, an earlier review conducted on the efficacy of rTMS in bipolar disorders demonstrated that rTMS over the right-DLPFC was deemed effective in the management of bipolar depression symptoms compared with sham [46]. This result is consistent with our findings, where the two studies [56,59] that applied rTMS over the R-DLPFC yielded some consistent positive outcomes in the symptoms of bipolar disorder among the study participants. Though the majority of the studies (six out of nine) targeted rTMS to the L-DLPFC, there does not seem to be any superiority between the left and right-DLPFC rTMS application in the management of bipolar disorder.

Furthermore, the long-term efficacy after acute augmentative rTMS treatment in patients with bipolar depression was tested in a 1-year follow-up study of 11 patients [59]. After 1 year of follow-up, the study demonstrated that the attainment of remission after acute rTMS treatment was predictive of response at 1 year. However, the absence of acute rTMS response predicted the absence of subsequent response in the long term.

Several elements may have accounted for the varying effectiveness of the rTMS intervention across the major domains of bipolar disorder. For example, the low sample sizes (n = 11 to 76) were too small of a sample from which to draw a definite conclusion. Secondly, a very important factor may be that the rTMS treatment was delivered at different phases of the bipolar illness and targeted at different clinical symptoms. This would strongly affect the clinical outcomes and efficacy.

Thirdly, the varying measurement scales used in evaluating similar outcomes makes it difficult to compare and evaluate the results against similar study findings. It is thus difficult to tell which rTMS protocols lead to the most significant outcomes and treatment response in bipolar disorder. However, owing to the comorbid nature and presentation of bipolar disorder, it may seem unrealistic to think of an optimal and standardized rTMS protocol that may work across domains of the different comorbidities even if they are made to target similar symptoms.

A further observation is the lack of data on the long-term and time-course effectiveness of rTMS treatment. Most studies conducted in the application of rTMS intervention usually evaluate the treatment outcomes at the acute phase or immediately after the final sessions of the treatment. In some special instances, the evaluation extends to a few months after treatment. However, only a handful of studies assess the effectiveness of rTMS treatment beyond these periods [59]. Evaluating the long-term efficacy of rTMS intervention in patients with bipolar is essential considering the debilitating, chronic, and high prevalent profile of this condition. Therefore, it will be of high research value to assess the sustainability of the effectiveness of rTMS treatment, most importantly evaluating maintenance strategies following remissions with rTMS treatment.

### 5.3. Tolerability/Side Effects of rTMS

The general essence of any treatment modality must address its effectiveness, safety, and tolerability concerns. rTMS treatment is noted in the literature for its safety and tolerability, with mild side effects in the patients to whom they are applied. The findings from this scoping review suggests that the rTMS intervention was safe and tolerated, and presented with minor side effects such as headache, dizziness, localized scalp pain, and stimulation of facial nerves, which disappeared soon after the application. Finally, neurocognitive processes can be enhanced by rTMS treatment in bipolar disorder patients in remission. rTMS treatment is deemed relatively simple to administer, safe, tolerable, and an effective way to manage cognitive impairments in patients with bipolar disorders. Regardless of the evidence in rTMS intervention having the potential to produce clinical significance in the treatment of bipolar disorders, the pathophysiology and clinical complexities of bipolar depression remain an area in need of further exploration for more accurate treatment protocols. More studies are thus needed in the area of rTMS to determine the specifics in stimulation parameters, effective treatment durations, and the brain region with the most significant effect.

## 6. Limitations

Finally, this review acknowledges some limitations worthy of mention. Firstly, our search strategy considered only English articles, hence we may have missed important studies published in other languages. Again, though every effort was made through our comprehensive search strategy to identify all relevant articles that met our inclusion criteria, we still might have possibly missed some important studies without knowledge. Therefore, while the data evaluated suggest that rTMS has the potential to be a clinically significant and effective therapeutic intervention for the management of the symptoms of bipolar disorders, more robust randomized controlled trials with higher sample sizes, longer treatment durations, and better stimulation parameters need to be conducted before a firmer conclusion can be drawn. Again, the heterogeneity of the bipolar spectrum disorder and the fact that each study targeted different phases of the illness make it difficult to generalize the outcome.

## 7. Conclusions

Despite the varying effectiveness, clinical viability, and outcomes demonstrated by the reviewed studies, there is enough evidence in support of the fact that rTMS treatment is a promising intervention for the management of bipolar disorders. However, to be able to draw conclusions on the efficacy of this treatment technique, more studies with well-defined stimulation protocols must be undertaken with larger sample sizes in the future.

## Figures and Tables

**Table 1:** Medline search strategy.

Search Strategy	Results
exp *Stress Disorders, Post-Traumatic/or (PTSD or ((posttraumatic or post traumatic or combat or war or trauma*) adj1 (stress* or neurosis or neuroses or nightmare*)) or ((traumatic or acute) adj (stress disorder* or stress symptom*)) or shell shock* or shellshock*).mp.	46,596
exp obsessive-compulsive disorder/or bipolar disorder	54,776
(Bipolar or bi-polar or manic-depress* or mania or obsessive-compulsive disorder* or OCD).mp.	102,961
1 or 2 or 3	147,991
Transcranial Magnetic Stimulation/	11,653
(Repetitive transcranial magnetic stimulation or rTMS).mp.	5423
5 or 6	13,372
4 and 7	492
